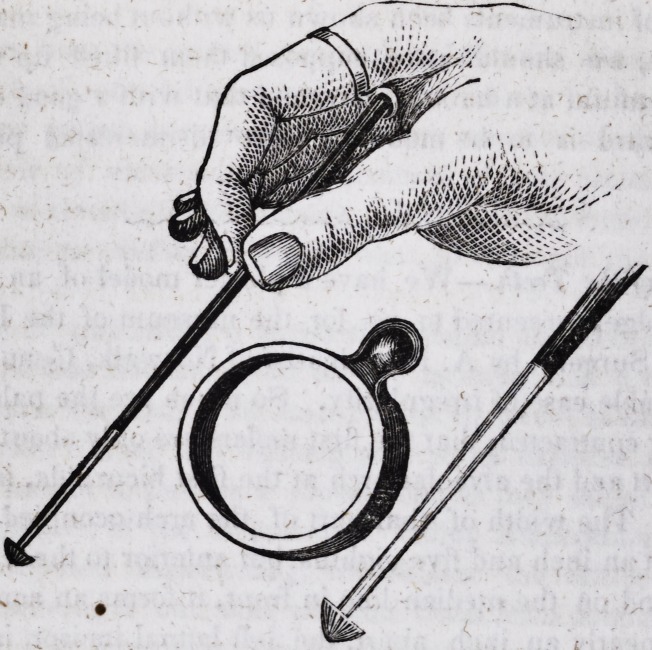# Dental Ring and Drill Socket

**Published:** 1846-12

**Authors:** 


*Dental Ring and Drill Socket.*—The above cut represents a very ingenious contrivance, invented by Prof. A. Westcott, for supporting and keeping in place the common burr drill, used in excavating cavities. It consists of a ring to be worn on the fore or middle finger, to which is attached a cup or socket, to receive the upper end of the drill, and to prevent it from hurting the hand. Various contrivances have been used for this purpose, and among the most effectual is a drill stock consisting of two portions, the part resting upon the hand being stationary, while that portion of it holding the drill is made to revolve.

The objection to this kind of stock is, that the drill has to be changed every time a different sized burr is required. But as this burr is an instrument very liable to be broken, and is one at best not long-lived, any stock requiring much time to adapt to it the drill, is particularly objectionable. The invention of Dr. Westcott wholly supersedes the necessity of fitting drills to a stock—the common cheap burrs are always ready for use, and even when the head is broken, its value is by no means destroyed, as it can be readily worked into an excavator. We have not used this kind of socket, but we have no doubt that it will greatly facilitate every operation where the burr can be used with advantage, and it will doubtless supersede every other contrivance for this purpose. The cup is partially filled with gold solder, which constitutes a surface not easily worn away, and one upon which the drill turns with great ease. Dr. W. informs us that he has used this contrivance for several months, and, to use his own language, "could no more dispense with it, than could the seamstress with her thimble."

*Balt. Ed.*

## Figures and Tables

**Figure f1:**